# Management of Feline Hyperthyroidism and the Need to Prevent Oxidative Stress: What Can We Learn from Human Research?

**DOI:** 10.3390/antiox10091496

**Published:** 2021-09-20

**Authors:** Alessia Candellone, Vittorio Saettone, Paola Badino, Flavia Girolami, Elisabetta Radice, Domenico Bergero, Rosangela Odore, Giorgia Meineri

**Affiliations:** 1 Department of Veterinary Sciences, School of Agriculture and Veterinary Medicine, University of Turin, Largo Braccini, 2, 10095 Grugliasco, Italy; vittorio.saettone@unito.it (V.S.); paola.badino@unito.it (P.B.); flavia.girolami@unito.it (F.G.); domenico.bergero@unito.it (D.B.); rosangela.odore@unito.it (R.O.); giorgia.meineri@unito.it (G.M.); 2 Department of Surgical Sciences, Medical School, University of Turin, Corso Dogliotti, 14, 10126 Torino, Italy; elisabetta.radice@unito.it

**Keywords:** feline hyperthyroidism, spontaneous animal model, thyroid, thyroid hormones, T3, T4, TSH, oxidative stress, methimazole, antioxidants

## Abstract

Feline hyperthyroidism is a clinical syndrome related to an excessive production of thyroid hormones, and it is considered as a spontaneous animal model for human thyrotoxicosis. Many shared features between the feline and the human disease have been identified so far, including pathogenesis, clinical signs, and treatment options. Although methimazole is considered the first-choice drug in both species, several side effects have been described. Furthermore, methimazole could interfere with the oxidative status, already perturbated by the disease. It has been reported in humans that dietary management, mainly through antioxidant supplementation, could mitigate this oxidative burden. The purpose of the review is to describe current therapeutic options in the course of feline hyperthyroidism and to summarize the state of the art relationship between dietary antioxidants administration and the reduction of methimazole side-effects in humans to support the use of this approach also in cats.

## 1. Introduction

The term “hyperthyroidism”, as described in humans, is an endocrine disorder where the thyroid gland produces excessive thyroid hormones. [1]. Similarly, feline hyperthyroidism (FHT) must also be considered as a multisystem disorder due to an increased production of thyroid hormones by the hyperfunctioning thyroid tissue. [2,3]. In addition, humans and cats share many common features of the disease such as clinical presentation and physiopathology. In cats, the disease most often results from benign adenomatous nodules of the thyroid tissue, making it like Plummer’s disease (toxic nodular goiter), while clinical and therapeutic management are comparable with the Basedow-Graves’ disease [4]. Metabolic oxidation has been correlated, both in hyperthyroid humans and cats, with signs and symptoms of the disease [5]; the decrease in antioxidant defenses and therefore the boost in oxidative stress can also be considered a risk factor for several idiosyncratic drug toxicity syndromes to anti-thyroid drugs, such as methimazole [6,7]. Achievement of euthyroidism results in normalization of redox markers [8,9,10], while the severity of clinical signs and the occurrence of methimazole-adverse reactions could be mitigated by the simultaneous administration of dietary supplements (e.g., antioxidants) [11,12,13]. The aim of this work is to (1) describe current therapeutic options in course of feline hyperthyroidism, with particular emphasis on nutritional management and (2) to summarize the state of the art relationship between the dietary supplementation of selected antioxidant molecules (such as curcumin, quercetin, resveratrol and vitamin E) and the reduction of methimazole side-effects in humans and to identify a possible rationale for this approach also in FHT.

## 2. Feline Hyperthyroidism

Feline hyperthyroidism is a disease in which there is an increase in the production and release of tetra-iodothyronine (T4) and triiodothyronine (T3) by the thyroid, with a suppressed thyroid stimulating hormone (TSH) [3].

### 2.1. Similarities with Human Disease

The domestic cat is the only animal species in which spontaneous hyperthyroidism related to senescence occurs frequently enough to allow a systemic investigation of its pathogenesis. Many common features have been highlighted between human and feline thyroid disease. In people, hyperthyroidism has been documented starting from 1913 by Plummer; he identified two different clinical presentations: the exophthalmic goiter (Graves’ disease, GD) and the toxic adenomatous goiter. In GD, increased T4 and T3 production was related to thyroid gland hyperplasia, whereas toxic adenomatous goiter was characterized by thyroid nodules and variable histological patterns. The latter disease involves the slow growth of autonomous functioning follicles. Toxic adenomatous goiter has been also described in hyperthyroid cats starting from 1979 by Peterson et al., and by two other reports by Holzworth, et al., in 1980 and Jones and Johnstone, in 1981 [14]. However, FHT is also similar to GD in its clinical appearance and therapy [4,15]. Indeed, restlessness, weight loss and tachycardia are shared physical abnormalities pharmacologically managed by the administration of the same molecules.

### 2.2. Etiology

In the 1980s, the first studies of FHT associated such a disease with a primary thyroid gland abnormality rather than with the effect of circulating TSH, or thyrotropin-releasing hormone (TRH). Indeed, when the thyroid tissue of hyperthyroid cats was experimentally transplanted subcutaneously into nude mice under TSH suppression, the thyroid cells maintained the cuboid shape, the high growth potential and functional autonomy [4,14]. In addition, Peterson et al., have described that a normal feline thyroid gland has subpopulations of follicular cells with high growth potential and TSH receptors with detectable basal constitutive activity [14]. Consequently, it is believed that cats destined to develop hyperthyroidism have subgroups of thyroid cells able to self-replicate and, if in adequate number, also to autonomously synthesize thyroid hormones. Scott-Moncrieff suggested that long-term stimulation of high growth potential cells make them autonomous from TSH feedback due to follicular cell mutations. Indeed, mutations with gain-of-function of the TSH receptor or of the alpha subunit of stimulating G proteins have been described in humans [4,14]. Eleven TSH receptor mutations were detected in 134 hyperplastic nodules of 50 hyperthyroid cats; five of them were also identified in thyroid tissue samples collected from hyperthyroid humans. Interestingly, out of the 41 cats with more than one nodule, 14 had nodules with different mutations [14]. A study conducted on thyroid adenomas of hyperthyroid cats identified a reduced amount of an inhibitory G protein as well [4,14]. A further study by Ward et al., suggested that decreased expression of certain inhibitory G proteins subtypes contributes to the molecular pathogenesis of FHT, rather than changes in TSH-stimulated G protein activity. Merryman et al., reported an overexpression of the oncogene product c-Ras in thyroidal nodular hyperplasia/adenoma tissues from 18 hyperthyroid cats [14]. Taken together, such evidence suggests that the multiple mutations in thyroid follicular cells may ultimately lead to thyroidal cells autonomy. Nonetheless, the etiopathogenetic mechanism underlying these mutations remains unclear, as is the reason why feline clinical overt hyperthyroidism has become much more common in the last 40 years, enough to make it the most frequent endocrine pathology in the geriatric age [14,15,16].

### 2.3. Pathogenesis

Primary thyroid disorders are almost always considered as the main cause of feline thyrotoxicosis. More specifically, hyperthyroid cats are more frequently diagnosed with follicular cell adenoma and multinodular adenomatous hyperplasia. Both are considered benign lesions and can occur simultaneously in the same subject. From the histological point of view, the follicular cells are uniform and cuboidal to columnar in shape with occasional papillary formations, circumscribing secreting follicles [4,14]. Thyroid adenomas are clearly identifiable by a thin fibrous capsule, sometimes invading the normo-functional tissue. In adenomatous thyroid hyperplasia, hyperplastic cells are organized in nodules and can vary in size, from 1 mm to more than 3 cm. Hyperplasia and adenomas are present in about two thirds of hyperthyroid cats [3]. Malignant neoplasm can affect one or both thyroid lobes and occurs in 1–3% of cats with FHT. Macroscopically, tumors can be encapsulated, mobile and clinically scarcely distinguishable from benign neoformations. Moreover, masses can be locally invasive, attached to the surrounding tissues and metastasizing to the tributary lymph nodes [17]. Feline thyroid cancers are generally well-differentiated adenocarcinomas composed of a uniform pattern of small follicles containing varying colloid amounts. Mixed compact and follicular morphological patterns are the most common, although primitive follicular and papillary patterns have also been described [15]. TSH secreting pituitary adenoma is a rare cause of hyperthyroidism in humans [18], but not yet documented in cats. Hyperthyroidism following iatrogenic ingestion of thyroid hormone, already diagnosed in dog fed raw diets, has not been described in cats yet [19].

### 2.4. Clinical Features

#### 2.4.1. Signalment

The mean age of onset of FHT is 13 years; however, a small number of cases are described under 4 years of age [14,20]. Several studies describe a greater genetic predisposition in breeds, such as the Siamese and the Himalayan, while a difference in incidence related to sex has not been demonstrated yet.

#### 2.4.2. Clinical Presentation and Laboratory Abnormalities

The disease is sneaky and progressive, and the severity of symptoms, along with the stage of the disease at the time of diagnosis depends on the amount of produced hormones. The most frequent clinical presentation of FHT is a middle-aged or senior, thin (98%), hungry (81%), restless (76%), tachycardic (66%) and polyuric (60%) cat. Furthermore, the patient has an anxious and sometimes aggressive behavior, with a reduced tolerance to any source of external stress [21,22]. In an older cat, weight loss, often coupled with an increased appetite, may arouse a reasonable suspect of hyperthyroidism. However, in a small number of cases, clinical signs may differ and can be difficult to hook to FHT. More specifically, “apathetic hyperthyroidism”, can represent the final evolution of the disease, capable of leading to heart failure. In severely affected patients, stress and restraint may also cause a sudden elevation of plasma T4 levels, and they can be considered possible precipitating factors for the so called “thyroid storm”, a term used for a rare clinical entity in humans. The multisystemic effects of thyroid hormone excess could also trigger a series of biochemical modifications and abnormalities. Some abnormalities, such as elevated plasma concentrations of liver enzymes and increased ratio of urinary corticoids to creatinine, can be managed with antithyroid drug therapy. Hemodynamic changes in FHT predispose to marked increases in glomerular filtration rate, glomerular hypertension, and hyperfiltration, often leading to mild proteinuria. Systemic arterial hypertension also occurs in 10 to 23% of hyperthyroid cats at the time of diagnosis, moreover 1 out of 4 patients initially classified as normotensive may became hypertensive during treatment. Etiopathogenesis of secondary hypertension in a course of FHT, however, is still debated. Publications in other species suggest that elevated thyroid hormones may increase cardiac sensitivity to circulating catecholamines and may also have a direct effect on heart cells [23]. One other major concern is the increase in plasma creatinine concentration following hyperthyroidism treatment. The endocrine disease may indeed mask the diagnosis of underlying chronic kidney disease (CKD), as the glomerular filtration rate (GFR) increases, and may be associated with loss of muscle mass; these two events may decrease serum creatinine concentration. Additionally, many hyperthyroid cats with concomitant CKD exhibit azotemia when GFR and body composition revert to euthyroid states. Currently, no clinical study can exactly predict which hyperthyroid cats have also a concurrently masked CKD. Identification of these cats could allow an earliest intervention, influence the therapeutic choice for the treatment of hyperthyroidism and potentially predict the onset of a post-treatment iatrogenic hypothyroid state. In hyperthyroid cats, symmetric dimethylarginine (SDMA) [24], together with urinary isoprostanes (renal oxidative stress markers) [25] could be used as early-stage kidney damage biomarkers. In a study from Peterson et al. (2018) [24], pretreatment SDMA concentration (>14 μg/dL) was high only in 14 of 42 hyperthyroid feline patients that developed azotemic CKD after treatment. SDMA test specificity as a predictor of masked azotemia was of 97.7% with a low sensitivity (33.3%). Although the SDMA concentration was insensitive, it had higher sensitivity than serum creatinine, even when the cutoff value for this parameter was set well below the upper limit of the reference interval. Lowering the upper cutoff for serum SDMA concentration from >14 μg/dL to >12 μg/dL resulted in a two-fold increase in test sensitivity, with only a slight decrease in specificity. [24].

#### 2.4.3. Diagnosis

When clinical history and biochemical abnormalities are suggestive of FHT diagnosis, a thorough palpation of the neck region is recommended. In fact, when the thyroid glands are enlarged, they descend along the trachea, sometimes reaching the thoracic inlet. Palpation is a specific examination to detect abnormalities in the shape of one or both lobes, and up to 90% of the changes can be detected in cats with hyperthyroidism [22]. Rarely, thyroid enlargement results from the presence of ectopic (sometimes intrathoracic) thyroid tissue. The final diagnosis should be based on direct measurement of thyroid function: the initial screening test is usually the basal serum concentration of T4. Reference values for total T4 are between 1 and 4.5 μg/dL in healthy cats. In a study on 917 untreated hyperthyroid feline patients, 221 ill cats suffering from non-thyroid disease and 172 clinically and biochemically healthy cats, 91% of the hyperthyroid subjects had elevated serum T4, while none of the cats unaffected by thyroid disease had a total T4 above normal limits. [26]. Today, the inclusion of T4 in many profiles for geriatric cat facilitates the diagnosis before the disease becomes clinically evident. Such screening approach is appropriate for the diagnosis of FHT due to its high specificity; however, it should be remembered that the serum concentration of T4 can also be influenced by non-thyroid diseases and alterations in metabolism that interfere with hormones binding to plasma carrier proteins, transport into cells, etc. In fact, according to Scott-Moncrieff, the serum concentration of fT4, measured by equilibrium dialysis, is a more effective parameter than the evaluation of the total concentration of T4 in elderly hyperthyroid cats with concomitant geriatric diseases, such as CKD [14]. However, Rijnberk et al. stated that “in most cases, measurement of fT4 concentration by direct equilibrium dialysis adds little or no useful diagnostic information”. “Non-thyroid diseases can be associated with false positive results and therefore feline hyperthyroidism should not be diagnosed only when a high concentration of fT4 is found” [22]. To date, the debate on the usefulness of determining fT4 in feline thyroid dysfunction is still widely open. Cats showing elevated fT4 and concentration of total T4 within normal limits should undergo other tests (e.g., scintigraphy) to confirm hyperthyroidism. Measurement of serum T3 concentration is scarcely used to diagnose FHT, while serum TSH concentration may have a clinical utility in selected cases. As effectively described by Peterson et al., serum TSH concentrations are abated in 98% of hyperthyroid cats, but concentrations are measurable in a few cats with mild to moderate hyperthyroidism [27]. For this reason, the measurement of serum TSH should be considered a very sensitive but not specific test for the diagnosis of FHT. Although the radioactive iodine uptake test with 131I or 123I is not always available, it could provide an effective contribution to the diagnosis [22].

## 3. Treatment Options of Feline Hyperthyroidism

Guidelines for FHT treatment have been recently summarized by Carney et al. (2016), according to currently available evidence-based knowledge [16]. The present review is written in line with this publication.

Principal treatment options for FHT, according to [16] are: radioiodine ablation of the thyroid (radioactive iodine therapy); surgical thyroidectomy; inhibition of secretion by antithyroid drugs; and administration of iodine-limited diets. The first two therapeutic alternatives are beyond the scope of the present review, thus only a brief summary of their pro and cons will be proposed. A thorough assessment of the pharmacological and dietary approaches will be presented.

### 3.1. Radioiodine Ablation of the Thyroid (Radio-Iodine Therapy) and Surgical Thyroidectomy

Radioiodine therapy has been recommended as the elective treatment [16,28]; however, it must be considered that, at present, this therapy is offered only by a limited number of facilities in some countries; furthermore, the confinement period up to 4 weeks after the injection of the radioactive compound is considered a limiting factor by the owner.

Surgical thyroidectomy, on the other hand, holds the risk of a general anesthesia in a geriatric and often unstable patient. Post-surgical complications, such as hypoparathyroidism or persistent hyperthyroid status due to ectopic thyroid tissue excreting thyroid hormones, contribute to reduce the number of patients that annually undergo this kind of procedure [16,28].

### 3.2. Antithyroid Drugs

Antithyroid drugs have two main purposes: short-term treatment, generally indicated to stabilize the patient before surgery, and long-term treatment, indicated to mitigate thyrotoxicosis. Furthermore, a trial with antithyroid drugs before radioiodine therapy or bilateral thyroidectomy can predict the risk of renal damage after definitive therapy [14,16,28]. Two molecules are available as veterinary drugs for the treatment of FHT: methimazole (MMI) and carbimazole (CMZ). In humans, MMI blocks thyroid peroxidase, thus preventing the synthesis of thyroid hormones. It is hypothesized that MMI accumulates, in the same way, in the thyroid glands of cats. Attack therapy of MMI should be an oral dose of 2.5–5.0 mg per cat twice daily. Twice-daily dosing is associated with less severe side effects than a higher once-daily dose [28]. However, once the cat is stabilized, administering the total daily dose every 24 h could increase owner compliance while maintaining euthyroidism. Most hyperthyroid cats have T4 levels within the reference range within 2–3 weeks from the starting of the treatment with antithyroid drugs [16,28]. Thus, the monitoring of T4 is recommended after that interval [28]. If the cat is still hyperthyroid, MMI dose adjustment can be accomplished with 1.25–2.5 mg/day increments until euthyroidism is achieved [28]. When maintenance doses >20 mg/day are needed, owner compliance should be questioned. If T4 falls below the reference range, MMI dosage should be reduced in steps of 1.25–2.5 mg/day and T4 and renal parameters should be re-checked after 1–2 weeks [28]. Unfortunately, oral anti-thyroid drugs are not free from side-effects. More to the point, administering a pill to a cat, once or twice daily for a life-long period, can be tricky and waste money [11]. Transdermal application of a pluronic-lecithin organogel (PLO) containing MMI has been suggested as an alternative treatment regimen; Hill et al. [29] recently demonstrated that a novel lipophilic formulation was as effective and safe as twice daily oral CMZ in the treatment of cats with hyperthyroidism. There are several advantages of the transdermal therapy over the oral therapy. The first one is represented by the possibility of avoiding the gastric route, reducing the potential for both degradation of the drug and gastric irritation. Side effects different from gastro-intestinal signs seem to be less severe and, according to the long-term follow-up study from Boretti et al., they do not occur during the first 3 months of treatment, as usually expected, but after a prolonged use of the gel. The second benefit is the opportunity of a more accurate dose adjustment [30]. The gel, in fact, is usually dispensed in 1ml (=100 IU) syringes without needle, with a minimum interval of 0.01 mL or 0.02 mL (=1IU/2IU) on the graduated scale. The MMI concentration varies from 2.5 mg/0.1 mL to 5 mg/0.1 mL; thus, the dose can be corrected by 0.125 mg/0.5 mg, which are hardly conceivable with tablets. Moreover, according to the manufacturer, tablets should not be divided. A further advantage is the non-invasiveness of the procedure and the improved owner compliance with the drug administration. Referring to possible disadvantages, the first is the absence of a PLO-based MMI available as a pharmacologically registered product; secondly, studies have shown a variable bioavailability of MMI in the form of PLO-gel, given as a single transdermal dose in healthy cats, despite a good clinical short-term response in sick cats have already been documented. Regional variations in percutaneous absorption, licking of the gel during grooming activity and evidence of trans-pinnal movement of MMI in a lipophilic vehicle applied to the inner ear in an in vitro model, may also have efficacy and safety implications for both cats and their owners. In a clinical trial performed by the author A.C. on 21 hyperthyroid cats evaluating efficacy, safety and owner compliance during transdermal MMI treatment, it emerged that it is an effective and safe administration option, at least for the short-term period, with an excellent owner satisfaction [29,30, Candellone et al., unpublished data]. As for the documented side effects of MMI [31,32,33,34,35], the most common ones concern the gastrointestinal tract, due to the possible irritating effect of oral MMI on the gastric mucosa, and the facial excoriation, likely associated with vasculitis. Instead, the most serious but rare disorders are liver disease and marked blood dyscrasias (severe leukopenia, anemia and/or thrombocytopenia). Moreover, other possible but uncommon complaints are lethargy and neuromuscular weakness, along with positive antibody titers to the acetylcholine receptor, due to acquired myasthenia gravis. Please see Table 1 for a more exhaustive list of suspected adverse drug reaction to oral and transdermal MMI. It has not yet been possible to demonstrate any relationship between MMI dosing regimen (once versus twice daily) and side effects. It should be noted that most side effects begin within the first 4–6 weeks of therapy and become less common after 2–3 months of starting treatment [28].

### 3.3. Administration of Iodine-Limited Diets

The administration of iodine-reduced diets is generally considered a valid alternative for the long-term management of subjects who cannot face a resolutive therapy. In fact, the reduction of iodine taken with the diet lowers the production of thyroid hormones, even if it does not always obtain an effective attenuation of clinical signs [36,37,38]. The utility of diets at different iodine concentrations from 0.15 mg/kg to 1.9 mg/kg on a dry matter basis (DMB) were investigated in two reports enrolling 33 hyperthyroid cats [39,40]. All cats were consuming commercial diets containing 1.9 mg/kg of iodine at the time of the diagnosis of hyperthyroidism. Subjects who ate a diet with a reduced iodine content of 0.17 mg/kg showed normal T4 values within 8 weeks, while cats who ate a diet containing iodine 0.28 mg/kg became euthyroid by 12 weeks. Conversely, the number of cats that became euthyroid was lower when they ate diets containing higher iodine concentrations (0.39 mg/kg [7 out of 9 cats] or 0.47 mg/kg [4 out of 5 cats]). More extensive thyroid profiles were also performed in 14 of 33 cats; concentrations of fT4, total T3, free T3 and TSH were within normal limits during treatment with iodine-restricted feed. If cats receiving a low-iodine diet do not become euthyroid within 4–8 weeks, it is advised to check for other sources of iodine-rich food accidentally given, including tap water, medications, or supplements [15,16]. Dietary therapy of feline FHT doesn’t have a human counterpart as low iodine diets are used only for a short time in human medicine and for special diagnostic purposes (e.g., prior to imaging for extra-thyroid metastases). In fact, iodine is also known to have antioxidant and anti-inflammatory properties, as well as a role in the prevention of breast cancer and fibrocystic breast disease [41]. One concern is that cats long-term managed with low-iodine diets may clinically develop goiter due to hyperplastic follicular cells and initiate the transformation from adenomatous nodules to thyroid cancer. In a report of eight cats with thyroid cancer, two cases had both adenomatous and carcinomatous cells contained in the same gland, suggesting that the carcinoma derived from a previous benign neoplasm [42]. Other concerns are related to the poor palatability of the diet in exigent pets and the possible nutritional inadequacy of such a long-lasting regimen for geriatric patients that have specific nutritional requirements [15,37,38].

Advantages and disadvantages of the four described treatment options are summarized in Table 2.

## 4. Mechanisms of MMI-Induced Organ Injury

**Figure 1:**
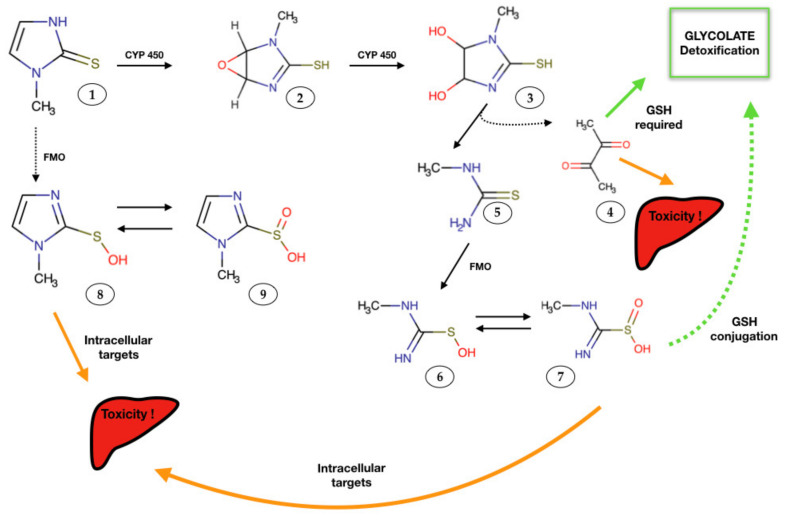
Proposed methimazole (MMI) metabolites, and their role in hepatic injury. Reactive intermediates formed during MMI metabolism may bound to macromolecule targets (e.g, proteins), and cause toxicity or might be detoxified by nucleophilic molecules such as glutathione [33,34]. FMO: Flavine-containing monooxygenase, CYP 450: cytochrome P450, GSH: reduced glutathione. Circled numbers: 1: methimazole; 2: epoxide metabolite; 3: dihydrodiol molecule; 4: glyoxal; 5: N-methyl thiourea; 6: sulfenic acid; 7: sulfinic acid; 8 and 9: other sulfenic and sulfinic acid species.

Possible manifestations of drug-induced damage must be taken into account when setting up a specific pharmacological therapy. In particular, hepatotoxicity, nephrotoxicity, vasculitis and blood dyscrasia represent possible clinical complications of antithyroid drugs used in the treatment of human and feline thyrotoxicosis [28,30,31,33]. As already mentioned above, MMI is the most used drug against hyperthyroidism. MMI-organ damage has been reported, with liver representing the main target. The etiopathogenetic mechanism of hepatic damage has not been precisely described yet; however, it is thought that mitochondrial toxicity caused by an oxidative stress state can trigger cell death and apoptotic phenomena in hepatocytes. The main enzymatic superfamilies that metabolize MMI are the haemoproteins (cytochrome P450, CYP 450) and the oxidoreductases, specifically flavin monooxygenase (FMO). The proposed pathways of bioactivation of MMI in the liver have already been explained in previous studies [33,34,35] and is here summarized in Figure 1.

Recently, Niknahad et al. studied the effect of MMI on hepatocyte mitochondria in two different models (in vitro and in vivo). The results of this study are controversial. In vivo, MMI caused mitochondrial damage in mice. Instead, in vitro MMI not only did not cause any mitochondrial damage, but also protect this organelle when isolated liver mitochondria were treated with different drug concentrations [32]. However, it should be considered that the observed differences may be associated with the drug bioactivation and the formation of reactive metabolites in vivo vs in vitro; in fact, biotransformation enzymes are absent in isolated mitochondria. In previous experiments, Heidari et al. found that some enzyme inhibitors effectively mitigated MMI-induced cytotoxicity in isolated hepatocytes. On the other hand, enzyme induction markedly increased MMI hepatotoxicity [33]. Thus, it becomes clear the importance of reactive metabolites, which must be considered as pro-oxidizing agents, in MMI-induced mitochondrial and subsequent hepatic dysfunction. Another investigation has indicated the radical elimination properties of MMI [32]. This study focused on the effect of MMI on IFN-alpha action in FRTL-5 rat thyroid cells. The results showed that IFN-alpha produced a significant amount of H2O2 in thyroid cells, inducing the tyrosine phosphorylation of STAT1 and STAT3. Furthermore, Kim et al. showed that MMI was able to clear H_2_O_2_ by one electron reduction, thus preventing reversible physiological inactivation of phosphatases in response to IFN-alpha signaling [34]. The net effect was therefore that MMI inhibited full activation of the JAK/STAT signaling pathway in FRTL-5 thyroid cells.

Taken together, these data suggest a paradoxical effect of MMI in different in vitro and in vivo models and further studies are needed to elucidate the mechanism of MMI-induced damage. Additionally, some studies have also suggested a variety of other factors that could affect drug-induced damage and may not be directly linked to drug metabolism. Among these, immunological reactions are the best known. An intriguing theory for immune-mediated drug-induced organ damage is the “haptenic hypothesis” [35]. Consistently with this theory, the reactive metabolites of the drug undergo a covalent bond with different proteins. The drug-protein complex is then identified by the immune system that, when activated, could lead to toxicity.

## 5. Oxidative Stress and Thyroid Hormones

### 5.1. Free Radical Generation and Antioxidant Defenses

**Figure 2:**
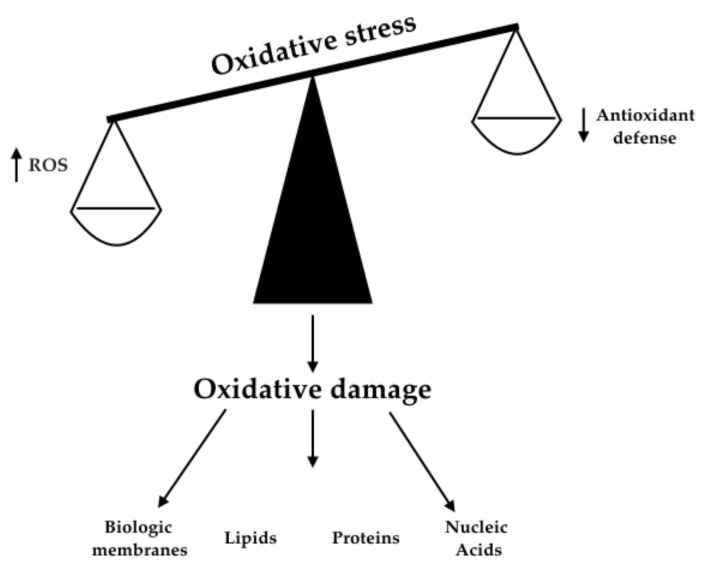
Mechanisms responsible for oxidative stress and cell damage [43,44,45].

Redox imbalance can be described as a disturbance of the balance between the production of free radicals (oxidative stress, OS) and antioxidant defenses, see Figure 2. Free oxygen radicals (ROS) are the most important in generating oxidative stress, and they include superoxide anion, hydrogen peroxide, hypochlorous acid and hydroxyl radical. The first three compounds are formed by the oxidation of energy substrates through the mitochondrial respiratory chain. Among ROS, the hydroxyl radical and the superoxide anion possess a high reactivity due to the presence of a single unpaired electron in the external orbital [43]. Double oxidases (DUOX) represent enzymatic steps for the generation of hydrogen peroxide and are also essential for the synthesis of the hormone catalyzed by thyroid peroxidase (TPO), [41]. Furthermore, NADPH oxidase 4 (NOX4) is a novel intracellular ROS generation system recently identified in the human thyroid [43,44,45,46]. Numerous endogenous defense processes against free radicals have been described in various cellular localizations (i.e., plasma membrane, mitochondria, peroxisomes and cytosol). Enzymes that stop or counter the production of free radicals are glutathione peroxidase (GPx), catalase (CAT), superoxide dismutase (SOD) and transition metal binding proteins, such as ceruloplasmin, ferritin and transferrin. SOD accelerates the process of dismutation of the superoxide anion in hydrogen peroxide and molecular oxygen. CAT detoxifies hydrogen peroxide by transforming it into molecular oxygen and water. GPx also participates in the detoxification of hydrogen peroxide when hydrogen peroxide levels are very high. Furthermore, GPx detoxifies the lipid peroxides by transforming them into the corresponding alcohols. Instead, the “scavenger” molecules, such as vitamin E, albumin, ascorbic acid, bilirubin, urates and thiols, stop the lipid peroxidation chain by neutralizing the intermediate radicals. The high diffusion speed of scavengers, together with their regenerative capacity, allows them to transform free radicals into more stable molecules [44].

### 5.2. Hyperthyroidism and Redox Imbalance

Hyperthyroidism is correlated with increased oxygen and intracellular ATP consumption, elevated ROS production and consequent dysfunction in the mitochondrial respiratory chain. Moreover, some studies underline the role of oxidative disturbances in the initiation of Graves’ disease (GD), Plummer’s disease and hyperthyroidism-induced damage, such as thyrotoxic myopathy, cardiomyopathy, and Graves’ orbitopathy (GO), [7,46,47,48,49]. However, some contradictions still emerge from a literature review related to studies conducted in animals [4,5,11]. Given the above, a summary of results deriving from clinical and experimental trials on hyperthyroid subjects, focused on evaluating their redox unbalance, will be presented in the following sections. Given that the more robust scientific literature has been published with regards to human patients, the first subchapter will be dedicated to human studies, then feline trials will be presented.

#### 5.2.1. Human Studies

In humans, most trials assessing the effect of thyroid diseases have been performed in patients diagnosed with GD, either under hyperthyroid condition or after restoration of euthyroid state. Untreated thyroidal hyperfunction status is associated with an increase of numerous intracellular and circulating parameters of OS [i.e., lipid peroxides, hydrogen peroxide, thiobarbituric acid-reacting substances (TBARS), determinable reactive oxygen metabolites (d-ROMs) and malondialdehyde (MDA)], as compared with basal concentrations detected under euthyroid state. In patients with overt hyperthyroidism, levels of circulating thyroid hormones correlate with concentrations of lipid peroxidation metabolites, but significantly higher ROS have also been observed in patients with a subclinical hyperfunction of the thyroid gland. A novel automated technique for the evaluation of the OS has been recently applied in hyperthyroid patients, confirming the existence of a redox burden [7]. Treatment with antithyroid drugs resulted in a declined concentration of OS markers, which may be related to the restoration of euthyroidism, as well as to the antioxidant properties of these molecules. Contradictory conclusions have been stated regarding the activity of the antioxidant defense system in human hyperthyroid cases [8,47,48,50]. Most trials have demonstrated an upregulation of antioxidant defense enzymes, as a homeostatic reaction to counteract the hyperthyroidism-induced ROS production. Komosinska-Vassev et al. [51] investigated selected markers of antioxidant status in 30 human patients with GD; they found an increase in the activity of some intracellular antioxidant enzymes (i.e., GPx, SOD and CAT), but not in serum GPx and total antioxidant status, as compared with age-matched controls. The authors postulate that the conflicting results between serum and erythrocyte antioxidant activities likely result in a quicker exhaustion of the antioxidant barrier. Bednarek et al. [9] measured the plasma antioxidant barrier in 47 GD cases of short duration (1–2 months), with and without GO, and found that CAT and SOD were increased when compared with healthy controls. Conversely, other studies have shown no evidence of increased antioxidant defenses in patients with untreated long-lasting hyperthyroid state [52]. The contradictory data on antioxidant barrier in patients with GD may be due, in addition to differences in assessment methods, to the duration of the hyperthyroid status at the time of evaluation. Indeed, in humans with short-lasting thyroid hyperfunction, we might expect an increment in the antioxidant defense mechanisms to balance the increased OS. On the other hand, in cases with a more prolonged duration of the hyperthyroid condition, the constant challenge of the antioxidant barrier system may account for the decreased serum and cellular antioxidant enzymes. Despite the above discrepancies, the administration of anti-thyroid drugs, able to restore the normal thyroid function, is usually also associated with the normalization of endogenous antioxidant defenses. A scavenging effect of MMI may also contribute to these adjustments [52] even if anti-thyroid drugs (ATD) could be responsible themselves of adverse-reactions via OS-induced damage (please see Section 4).

#### 5.2.2. Feline Studies

Feline studies evaluating OS in course of thyrotoxicosis are scattered [5,11,24]. In 2009 Viviano et al., [53] quantified erythrocyte reduced glutathione, plasma cysteine, and plasma ascorbate in clinically ill cats and dogs. Diseases were categorized based on illness severity assessed at the time of admission (mild, *n* = 19; moderate, *n* = 11; and severe, *n* = 7) and the “mild category” included two cats (2/37) diagnosed with FHT. Ill cats were characterized by higher ascorbate concentrations (10.65 μM, range 1.13–25.26) as compared to controls (3.68 μM, range 0.36–13.57; *p* = 0.0009), suggesting a possible unique species response to OS. Branter et al., for instance, assessed antioxidant status in hyperthyroid cats before and after radioiodine therapy [25]. Fifty-one client-owned hyperthyroid patients that presented for radioiodine treatment were recruited for the study. Both control and hyperthyroid cats were screened with a physical exam, cell blood count, biochemical panel, urine specific gravity (USG), blood pressure and serum total T4. Additional blood was also obtained for antioxidant assays (including measurement of plasma free vitamin A), along with urine for urinary free 8-isoprostane concentrations. Most of the blood screened antioxidants were not significantly different in hyperthyroid cats compared to controls, but plasma free vitamin A was higher in diseased animals. Similarly, urinary isoprostanes were increased in hyperthyroid cats compared to healthy subjects. Both abnormalities normalized after radioiodine treatment [25]. Candellone et al., [5] screened OS and antioxidant status markers in cats suffering from thyroidal and non-thyroidal diseases with respect to a control group of healthy subjects. Forty cats with untreated hyperthyroidism, 45 chronically ill cats with non-thyroidal illness, and 39 healthy cats were recruited for an observational cross-sectional study. Determinable reactive oxygen metabolites (d-ROMs) were used as oxidative stress markers. Antioxidant status was determined using the OXY-Adsorbent test to quantify the plasma barrier to oxidation. The Oxidative Stress index (OSi) was calculated as the ratio of d-ROMs and OXY-Adsorbent test values. The OXY-Adsorbent test results in hyperthyroid cats (265 ± 68 μmol HClO/mL) were significantly lower than those in healthy cats (390 ± 83 μmol HClO/mL; *p*  < 0.01) and chronically ill cats (306  ±  45 μmol HClO/mL, *p*  < 0.05). Moreover, the Osi value in hyperthyroid patients (0.8 ± 0.2 CarrU/μmol HClO/mL) was significantly higher (*p* < 0.001) than that of the healthy feline (0.3 ± 0.1 CarrU/μmol HClO/mL), [5].

## 6. Nutritional Management

Nutritional management of FHT could be aimed to pursue three different scopes: a therapeutic scope, a dietary scope and a synergistic scope.

As already discussed in Section 3.3, the administration of iodine-restricted commercial diets to hyperthyroid cats (therapeutic scope) is aimed to limit the synthesis of thyroid hormones, leading to euthyroidism in the majority of cats fed exclusively with this diet [36]. This approach represents the sole treatment option when other solutions (i.e., radio-iodine therapy, surgery, antithyroid drugs administration) are not feasible. However, the nutritional composition of the commercial iodine-restricted diet is not adequate to completely satisfy dietary requirements of the hyperthyroid patients as described in Table 3 [15,37,38]. Regardless of the therapeutic approach, a maintenance diet, iodine-unrestricted, but specifically formulated to satisfy nutritional requirements of a geriatric and hyperthyroid patient, could represent a useful tool to contrast metabolic changes that usually occur in course of thyrotoxicosis [15]. It should also be considered that dietary management can possibly exert a synergistic effect with the pharmacological treatment (synergistic scope). In fact, administration of dietary antioxidants to hyperthyroid patients receiving oral antithyroid drugs could possibly ameliorate their clinical outcome and reduce MMI-related side-effects, as already demonstrated in human and animal studies [5,11]. According to the authors, the pursuit of a dietary and synergistic scope plays a crucial role in the “multimodal approach to the hyperthyroid cat” that is proposed in the present review. In the following paragraphs, specific metabolic changes of the hyperthyroid patient will be analyzed, and the dietary (chapt. 6.1) and synergistic approaches (chapt. 6.2) will be discussed on the basis of the available literature with regards to in vivo and in vitro models.

### 6.1. Dietary Scope

Compared to omnivorous species, the cat is an obligatory carnivore. Thus, the protein requirement must be satisfied in the formulation of a diet for hyperthyroid cats: as previously described, in these subjects, muscle loss and nitrogen catabolism are a constant feature and proteins represent the main macronutrient for muscular mass homeostasis. Based on these considerations, Peterson et al., (2014) recommend that at least 40% of daily calories should be provided by protein, or greater than or equal to 12 g/100 kcal of metabolizable energy (ME) [15]. This recommendation remains unchanged once the euthyroid state is restored. As many cats become sarcopenic with age, feeding older cats with larger amounts of high-quality protein can slow down muscle mass loss [15,38,54]. Since a fair number of hyperthyroid cats have mild hyperglycemia (subclinical diabetes), a low carbohydrate dietary regimen is also strongly advised. To this end, a target of less than 15% of total calories from carbohydrates, or less than 4.5 g/100 kcal ME is indicated. Indeed, a low carbohydrate diet can prevent the development of overt diabetes after effective control of hyperthyroidism. The remaining part of the necessary calories must be guaranteed by lipids. For the underweight hyperthyroid cat, lipid caloric density can be helpful. However, after euthyroidism has been restored, calorie intake and consequently body weight and body condition score (BCS) should be routinely checked to maintain ideal conditions [15]. If hyperthyroid individuals also have CKD, a mild to moderate reduction in dietary phosphate is warranted, depending on the CKD stage, as assessed according to International Renal Interest Society (IRIS) guidelines [15,55]. Commercial maintenance diets contain 125–500 mg/100 kcal of phosphorus; in cats with CKD IRIS stage 1 or 2, Peterson et al., (2014) suggest a diet containing 125–250 mg of phosphate/100 kcal, which is necessary to maintain phosphatemia [15]. As described in Table 3, currently few commercial diets present an ideal composition to meet the needs of hyperthyroid cats. Other useful complementary foods for hyperthyroid patients are potassium gluconate, water-soluble B-complex vitamins, omega-3 fatty acids, and, as described below, antioxidants.

### 6.2. Synergistic Scope

#### 6.2.1. Antioxidant Supplementation

Antioxidant supplementation acts a synergistic scope in course of the pharmacological treatment. A customized nutrition to the hyperthyroid patient could be formulated not only to counteract metabolic effects of thyrotoxicosis, but also to improve efficacy and safety of the pharmacological treatment with antithyroid drugs, as already stated at the beginning of the present section. In vitro experiments and in vivo studies, performed on experimentally induced hyperthyroid laboratory animals, already demonstrated that the administration of antioxidants (mainly curcumin, quercetin, resveratrol and vitamin E) is able to counteract the OS-related organ damage induced by elevated thyroid hormones [5,7,9,11]. The choice of the four natural antioxidants mentioned above and contained in widely used fruit and vegetable products, was driven by two aspects. The first one was the abundancy of literature showing their efficacy in protecting animal cells and tissues from the OS injury induced by elevated T4 and/or MMI administration [8,13,56]. The second reason concerned the mechanism of action and the peculiar clinical effects of each of the selected compounds [57,58,59,60,61]. For example, curcumin is the most known hepatoprotective agent [58]; resveratrol is capable to improve cardiovascular status [58]; quercetin ameliorates intestinal absorption and bioavailability of both compounds [59,61], exert a renal protection and also inhibits thyroid type 1 deiodinase activity [62]; while vitamin E, the antioxidant par excellence, has a principal function in destroying peroxyl radicals, thus protecting polyunsaturated fatty acids biological membranes from oxidative damage. The concomitant supplementation of these compounds, at proper dosages, could possibly ameliorate clinical, biochemical and pharmacological outcome of hyperthyroid cats treated with MMI, which are at risk of developing hematological, cardiac, renal and hepatic alterations due to OS in course of the disease [11]. In the following section, mechanisms of actions and antioxidant effect of curcumin, quercetin, resveratrol, and vitamin E, administered alone or in a mixture, in thyroid cell lines or in hyperthyroid rats will be presented. Unfortunately, specie-specific published studies on feline in vitro-models are unavailable, but the authors already obtained preliminary data demonstrating the protective effect of quercetin and curcumin on methimazole and T3 induced oxidative stress in a feline kidney epithelial cell line (CRFK), (data not shown).

##### Curcumin

Curcumin is a natural phenolic compound isolated as a yellow pigment from turmeric (*Curcuma longa*), used as food coloring (E100) and spice. Furthermore, curcumin finds use in the Indian and Chinese medicine system. From a biochemical point of view, curcumin inhibits the inhibitory kappa B alpha kinase (IκBα kinase), a key activator of nuclear factor κB (NF-κB); such activity makes curcumin effetive in various metabolic endocrine states such as thyroid disease [58,60]. Additionally, dried turmeric rhizome has no toxicity, as evidenced by the fact that it has been authorized for human consumption and is widely used as a food seasoning [58]. The effects of curcumin supplementation on selected aspects of redox balance of hyperthyroid-induced animal models are summarized in Table 4.

##### Quercetin

Quercetin, a flavonoid contained in many fruits and plants, is used for food purposes in the formulation of many supplements. Quercetin has beneficial effects in inflammatory and immune conditions, as well as in thyroid disease. Biochemical variation in laboratory animals treated with thyroid hormones before and after supplementation with quercetin or its metabolites are summarized in Table 5.

##### Resveratrol

Resveratrol is a non-flavonoid phenol found in numerous plants such as red grapes (*Vitis vinifera*) and the Japanese knotweed (*Fallopia japonica*). Numerous in vivo and in vitro studies have documented the antioxidant, anti-inflammatory and anticarcinogenic capabilities of this compound. Moreover, recent experiments highlighted resveratrol anti-thyroid activity. These results are summarized in Table 6. All these data suggest that activities of resveratrol may vary in vitro based on the type of cells treated (normal vs cancerous thyroid cell lines). One possible explanation is the involvement of the aryl hydrocarbon receptor (AhR), of which resveratrol is modulator with agonist and antagonist activity in different cells [68,69]. Previous experiments have shown that AhR activation can cause alterations in thyroid function, with down-regulation of the expression of the NIS gene. Another potential mechanism of action behind the anti-thyroidal effect of resveratrol is the involvement of sirtuins. Resveratrol is an activator of SIRT1, a protein deacetylase that regulates the expression of several transcription factors and is widely expressed in most mammalian organs, including the thyroid [68,69].

##### Vitamin E

Vitamin E is the best-known antioxidant molecule. Tocotrienols and tocopherols, in fact, inhibit lipid peroxidation thanks to their capability to eliminate lipid peroxyl radicals faster than these radicals can react with the side chains of adjacent fatty acids [57]. Furthermore, vitamin E plays a protective role in the membrane by acting as a structural component. The structural “antioxidant” role of vitamin E may also be partly due to the downregulation of mitochondrial ROS generation; in fact, it has been demonstrated that α-tocopherol lowers lipid peroxidation and increases total antioxidant capacity in liver homogenates and hyperthyroid rat mitochondria [57,65,66]. Results of Vitamin E supplementation on selected oxidative parameters and antioxidant genes expression in hyperthyroid-induced animal models are summarized in Table 7.

#### 6.2.2. Efficacy of Antioxidant Administration

##### Human Studies

Human studies demonstrated that antioxidant treatment might be useful in those hyperthyroid patients with a need for rapid clinical improvement, such as danger of thyrotoxic crisis, cardiomyopathy and MMI intolerance [8,10]. Antioxidant supplementation might be also an indication before a therapeutic dose of radioactive-iodine (I131) administration or before thyroidectomy. Clearly, antioxidant therapy is not intended to replace conventional treatment of hyperthyroidism with antithyroid agents such as MMI, but it could be considered as a new therapeutic tool to improve the clinical manifestation of this illness, by mean of a multimodal approach. At the best of the author’s knowledge there are no clinical trials evaluating the efficacy of curcumin, resveratrol and quercetin, administered as single agents or in a mixture, in the course of human hyperthyroidism. Most of the studies evaluated the effects of vitamin E on clinical, biochemical and OS markers, during MMI-treatment or before radio-iodine therapy. In these trials, vitamin E is usually supplemented in combination with other compounds, which may also exert antioxidant properties. Of particular interest is a trial from Guerra et al., [8] were an antioxidant mixture (LAROTABE ^®^, containing vitamin E 200mg, b-carotene 3 mg, vitamin C 250 mg, Cu 1 mg, Zn 7.5 mg, Mn 1.5 mg, Se 15 ug) was evaluated in the treatment of Graves’ disease (GD) in 56 hyperthyroid patients. Patients were divided into three groups: group A, receiving MMI alone, group B, receiving LAROTABE^®^ alone and group C receiving both products. Clinical scores, thyroid hormones fluctuations, activities of antioxidant enzymes and OS markers (plasma MDA) were monitored during an 8 week period. When hyperthyroid patients were treated with MMI alone (group A), they became clinically and biochemically euthyroid after approximately 8 weeks. Antioxidant enzymes normalized their values, and plasma MDA decreased to similar levels of euthyroid controls. When hyperthyroid patients were treated with antioxidants alone (group B), a significant decrease of the standardized clinical score was observed after 4 weeks [8]. The activities of antioxidant enzymes improved, and MDA values returned to normal, even if thyroid hormones were still above the reference range. The combination of MMI and the antioxidant mixture (group C) shortened the period of normalization of the clinical score, as well as the normalization of hormonal concentrations from approximately 8 to 4 weeks, when compared with the administration of MMI alone. According to these results, treatment of hyperthyroidism with antioxidants rapidly improved the patients clinically, even in the presence of high concentrations of thyroid hormones. It also appeared to potentiate the effects of MMI on the synthesis of thyroid hormones. It could be postulated that elevated thyroid hormones produce signs and symptoms of hyperthyroidism through an increase in free radicals as it has been shown in GD [7,43]. When such augmentation is neutralized with antioxidants, the patients improve clinically, although this treatment has no effect on the production of thyroid hormones. Authors did not have a clear explanation about the increased effect of MMI when administered together with the antioxidant mixture. Antioxidants could interfere with the action of the peroxidase in one or both reactions, iodide oxidation and/or coupling reaction. As seen in Section 3, in fact, MMI acts by inhibiting the peroxidase; in this way a synergetic result could be obtained. However, an inconsistent justification was found for the lack of effect that antioxidant mixture had on the levels of thyroid hormones when administered without MMI, and it can be speculated on the possibility of a dose too low to inhibit thyroidal oxidations and hormonal synthesis. Given the above, antioxidants appear to be an effective coadjutant treatment of hyperthyroidism in humans, as they induce a rapid clinical improvement, evident as soon as 4 weeks after their administration [8].

##### Feline Studies

Up to date, there is only one study from Candellone et al. [11] evaluating the effect of dietary antioxidants supplementation (curcumin, resveratrol, quercetin and vitamin E) in MMI-treated hyperthyroid cats. A series of hyperthyroid client-owned feline patients were randomly allocated in group M + A (MMI + antioxidants) or group M (MMI + placebo). Clinical findings, haemato-biochemical parameters, concentration of circulating thyroid hormones, selected markers of OS and antioxidant status [i.e., determinable reactive oxygen metabolites (dROMs), OXY-adsorbent test values, and oxidative stress index (OSi) values], and MMI-related adverse events were monitored. Group M had a significantly increase in dROMs and OSi values during the study. Likewise, OXY-absorbent test values were significantly higher in group M + A than in group M at the end of the trial. Moreover, the occurrence rate of MMI-adverse events in the group treated with antioxidants was lower than in the untreated cohort of cats. These preliminary results show that the dietary supplementation of antioxidants in hyperthyroid feline patients receiving ATD could exerts a protective effect against OS, likely contributing to the reduction of drug idiosyncrasies [11].

## 7. Conclusions

Hyperthyroidism in accompanied, both in humans and in animal models, by increased free radical formation. Anti-thyroid drugs, such as MMI, are able to restore euthyroidism in diseased feline patients, but they could be responsible for adverse reactions leading to organ damage and to a further increase in redox imbalance. Dietary antioxidant supplementation of molecules such vitamin E, curcumin, resveratrol and quercetin showed a protective effect, in vitro and in vivo trials, on MMI-induced tissue injury and on the amelioration of clinical signs related to thyrotoxicity. Correcting the state of impaired redox balance with a customized nutritional regimen in addition to traditional thyrostatic therapy could be then advised also while managing feline hyperthyroidism.

## Figures and Tables

**Table 1:** Suspected adverse drug reactions to oral and transdermal methimazole (modified from [28]).

Side-Effects	Dose Range (Total mg/day)	Administration	Frequency (%)	Time to Onset (Days)	Recommendation	Monitoring/Comments
Hepatopathy	5 to 15 5	Oral Transdermal	2.6 4	15 to 60 14	/	Liver enzymes function
Bleeding diathesis	5 to 15	Oral	2.5	15 to 50	/	In most cases associated with thrombo- cytopenia
Marked thrombocytopenia	5 to 20 10	Oral Transdermal	2.8 8	14 to 90 14 to 28	Discontinue treatment	Platelet counts 2 weeks later
Agranulocytosis neutropenia	5 to 20 5 to 10	Oral Transdermal	2.7 7	26 to 95 Within the first 4 weeks	/	cell blood count two weeks later
Myasthenia gravis	Unknown	Oral	4 cases reported	60 to 120	Discontinue treatment, administer steroids	
Anaemia	2.5 to 5	Oral	1 case report	After 3 years of treatment	Discontinue treatment if anaemia is severe, no re- challenge	cell blood count 2 weeks later
Gastro-intestinal upset or lethargy	2.5 to 15	Oral	Vomiting, nausea, 9.3 Anorexia, 8.9 Unspecified GI upset, 23 Lethargy, 10.5	7 to 60 1 to 78 Within the first 4 weeks 1 to 60	Continue treatment Lower dosage if no improvement	
Generalized peripheral lymphadenopathy	10	Oral	1 case report	Within 2 weeks	Discontinue treatment	
Antinuclear antibodies	2.5 to 20	Oral	23	10 to 870	Not routinely measured	
Mild haematological abnormalities	2.5 to 25	Oral	16.4	10 to 490	Continue treatment, unless associated with clinical signs	
Positive DAT (Direct Antiglobulin Test)	10 to 15	Oral	1.9	45 to 60	Continue treatment if no haemolytic anaemia	
Dermatological reactions (i.e., facial excoriation)	5 to 15 5 to 10	Oral Transdermal	4 8	6 to 40 Within the first 4 weeks	Ideally, discontinuation of treatment is recommended	

**Table 2:** Advantages and disadvantages of FHT treatment (modified from [14]).

Treatment	Advantages	Disadvantages
Radioactive Iodine	- Kills abnormal cells - Cure rate ≥95% - Rare side effects - Risk of permanent hypothyroidism - Elective treatment in humans	- Requires special license and facility - Hospitalization - Cat confined for minimum 2 weeks after discharge - Irreversible
Oral or transdermal medication	- Efficacy ≥95% - Easy to administer - No hospitalization	- Relapse when medication are stopped - Daily medication and frequent vet checks - Possible adverse reactions - Tumor may become malignant
Surgical thyroidectomy	- Efficacy ≥90% if both glands are removed - Efficacy 35–60% if one gland is removed	- General anesthesia - Possible parathyroid gland damage - Hospitalization - Irreversible
Diet	- Efficacy ≥82% while on diet. - Recommended for cats with CKD	- No other feed permitted - Relapse rate 100% when diet is discontinued

**Table 3:** Nutritional values of three feline commercial diet and comparison with recommendations from [14,15,16,54,55] and FEDIAF guidelines 2020 [56]. The dark red text identifies the closest value to Peterson’s recommendations for nutritional management of FHT.

	Nutritional Recommendations for FHT [14,15,16,54,55]	Restricted-Iodine Commercial Diet	Feline Senior Commercial Diet 1	Feline Senior Commercial Diet II	Nutritional Recommendations FEDIAF [56]
Kcal ME/100 g DMB	↑↑ caloric density/100 g DMB	450	412	390	100 kcal/kg
Protein % MER and/or g/100 g DMB	40% MER	34.07 g/100 g DMB 30.2% MER	30.4 g/100 DMB	39.1 g/100 DMB 40% MER	25 g/100 g DMB
Protein (g/100 kcal ME)	12	7.58	7.37	10	6.25
Lipid % MER and/or g/100 g DMB	40-50% MER	24.97 g/100 g DMB 49.9% MER	15.2 g/100 g DMB 33.2% MER	10.8 g/100 g DMB 24.9% MER	9
Lipid (g/100 kcal ME)	/	5.55	3.7	2.76	2.25
NFE % MER and/or g/100 g DMB	<15% MER	33.65 g/100 g DMB 29.6% MER	36.8 g/100 g DMB 35.7% MER	30.3 g/100 g DMB 31% MER	/
NFE (g/100 kcal EM)	/	7.48	8.9	7.74	/
Phos %	Depends on IRIS CKD stage	0.6	0.59	0.86	0.5
Phos (mg/100 kcal ME)	125–250 But depends on IRIS CKD stage	134.12	133.49	219.9	125

DMB: dry matter basis; ME: metabolizable energy; MER: metabolic energy requirements; NFE: nitrogen-free extract or carbohydrates, Phos: phosphorus.

**Table 4:** Curcumin effects on redox status of hyperthyroid-induced animal models.

Species	Organ	Investigated Aspect	Results	Reference
rat	cerebral cortex and cerebellum	lipid peroxidation and SOD activity	↓ to control values of elevated levels of lipid peroxidation ↑ activity of SOD and of the translated products of SOD1 and SOD2	[63]
rat	renal cortex	modulation of antioxidant enzymes (i.e., reduced GSH and ascorbic acid, SOD, CAT) and OS parameters (i.e., lipid peroxidation and protein carbonylation)	restored control values levels of OS parameters and small antioxidant molecules ↑ SOD and CAT	[64]
rat	liver	lipid peroxidation and antioxidant genes expression	alleviation of lipid peroxidation and up-regulation of antioxidant genes	[65,66,67]

CAT: catalase; SOD: superoxide dismutase; GSH: glutathione reductase.

**Table 5:** Effects of quercetin and rutin supplementation on biochemical and redox status of hyperthyroid-induced animal models.

Species	Antioxidant Compound Administered	Tested Effect	Results	Reference
mouse	quercetin	thyroid function, hepatic activity and peroxidation, antioxidant enzymes activity	↓ T3 and T4 levels; ↓ hepatic activity of glucose-6- phosphatase (G-6-Pase) 5 ‘-monodeiodinase and hepatic lipid peroxidation SOD and CAT activity	[59]
rat	rutin (glycoside of quercetin)	thyroid function, antioxidant enzymes activity	no changes in TSH; slight ↓ T3 and T4 levels; significant ↓ hepatic DIO1 activity ↑ in hypothalamic, pituitary and brown adipose tissues DIO2 activity	[61]
rat	rutin	activity of SLC5A5 and TSHR genes	up-regulation	[61]

CAT: catalase; SOD: superoxide dismutase; SLC5A5: solute carrier family 5 member 5; TSH: thyroid stimulating hormone; TSHR: thyroid stimulating hormone receptor; T3: triiodothyronine; T4: thyroxine.

**Table 6:** In vivo and in vitro effects of resveratrol treatment.

Model	Investigated Aspects	Results	Reference
in vitro (normal rat thyroid cells)	NIS gene expression and iodide uptake	Inhibition of both	[67]
in vitro (immortalized cell line of untransformed rat thyrocytes, FRTL-5)	NIS gene expression and iodide uptake	↓ sodium/iodide symporter rna and protein expression as a function of time; ↓ cellular iodide uptake	[68]
in vivo (Sprague-Dawley rat)	NIS gene expression and thyroid function	Inhibition of both	[68]

NIS: sodium/iodide symporter.

**Table 7:** Vitamin E effects on selected parameters of hyperthyroid-induced animal models.

Species	Organ	Investigated Effect	Results	References
rat	renal cortex	oxidative stress parameters (lipid peroxidation and protein carbonylation) and small antioxidant molecules (reduced glutathione and ascorbic acid); antioxidant enzymes activities (i.e., SOD, CAT); translated product of Cu/Zn-SOD, Mn-SOD	Restoration of oxidative stress parameters and small antioxidant molecules levels; ↓ in translated product of Cu/Zn-SOD, Mn-SOD and CAT	[64]
rat	liver	hepatic function; hepatic complexes I and II mediated respiration and hepatic oxidative stress (protein carbonylation)	amelioration of hepatic function; ↓ state 4 respiration of complex I; ↑ complex II respiration both at state 3 and state 4 level; ↓ protein carbonylation	[65]
rat	liver	antioxidant genes (AOG) expression	alleviated message levels of SOD and CAT; ameliorated GPx1 and GR mRNA levels; alleviate SOD1, CAT and GR translated products; normalization mitochondrial SOD1	[66]

AOG: antioxidant genes; CAT: catalase; SOD: superoxide dismutase.

## Abbreviations

ATD: anti-thyroid drugs; CAT: catalase; CKD: chronic kidney disease;CMZ: carbimazole; CYP 450: cytochrome P450; DMB: dry matter basis; d-ROMs: determinable reactive oxygen species; DUOX: double oxidase; FHT: feline hyperthyroidism; GD: Basedow-Graves’ disease; GFR: glomerular filtration rate; GO: Graves’ orbitopathy; GPx: glutathione peroxidase; GSH: reduced glutathione; MDA: malondialdehyde; ME: metabolizable energy; MMI: methimazole; OS: oxidative stress; Osi: oxidative stress index; SDMA: symmetric dimethylarginine; SOD: superoxide dismutase; T3: tri-iodothyronine; T4: tetra-iodothyronine or thyroxine; TBARS: thiobarbituric acid reactive substances; TPO: thyroid peroxidase; TSH: thyroid stimulating hormone.
